# The Influence of Digital Tools and Social Networks on the Digital Competence of University Students during COVID-19 Pandemic

**DOI:** 10.3390/ijerph18062835

**Published:** 2021-03-10

**Authors:** Javier Rodríguez-Moreno, Ana María Ortiz-Colón, Eulogio Cordón-Pozo, Miriam Agreda-Montoro

**Affiliations:** 1 Department of Pedagogy, Faculty of Humanities and Education Sciences, Campus Las Lagunillas, University of Jaen, 23071 Jaén, Spain; jrmoreno@ujaen.es (J.R.-M.); magreda@ujaen.es (M.A.-M.); 2 Department of Business Organization II, Faculty of Economics and Business, Campus La Cartuja, University of Granada, 18010 Granada, Spain; ecordon@ugr.es

**Keywords:** higher education, COVID-19, digital tools, social networks

## Abstract

The pandemic caused by COVID-19 has generated a transformation in students’ competences and university education, especially in the use of digital tools. This study aims to analyze the use of digital tools and social networks of university students during the COVID-19 pandemic. For the collection of information, a validated Likert questionnaire (10-point scale) was adopted. The instrument consisted of a total of 66 items comprising a total of seven dimensions. The sample contained 581 students pursuing degrees in Childhood Education and Primary Education. The analysis of the available information was carried out in two different stages. First, we started by performing an exploratory factorial analysis (EFA) to determine the underlying structure of the Digital Competence of Higher Education Students (DCHES) scale factor. In the second phase, we used SEM (structural equation modeling), a statistical approach to test the relationships between observed and latent variables. More specifically, we estimated a multiple indicators multiple causes (MIMIC) model. The results showed the importance of two of the considered covariates in explaining the variability of the different dimensions of the scale analyzed (DCHES) considering the use of social networks and digital tools of university students. In this sense, both the degree to which virtual tools are used to develop teamwork and the degree of use of YouTube when communicating most fully explained the level of digital skills among the university students studied.

## 1. Introduction

The pandemic caused by COVID-19 (Coronavirus Disease 2019) has had a major impact in all contexts, forcing university education to undergo great changes in a very short period of time, both for teachers and for students, in order to adapt itself to the new situation using the digital tools at its disposal. In this context, it is necessary to consider the competences that affect students, such as effective, emotional, and social competences, as students have been forced to work in unknown contexts featuring the exclusive use of digital platforms. Cabero [1,2] argues that the wellbeing of students includes five areas: material, social, physical, psychological, and cognitive wellbeing, although educational sociologists consider economic and social wellbeing to be an influencing factor for the rest.

In addition, Ng [3] considers digital literacy as “multiple literacies related to the use of digital technologies”, indicating that these technologies “are the subgroup of electronic technologies that include hardware and software used by people with educational, social or leisure purposes at school or at home”. This same approach establishes the concept of literacy as the result of “the intersection of three dimensions: the technical dimension, the cognitive dimension and the social–emotional dimension of digital literacy” (p. 1067).

In other words, we must ensure that the new educational programs of UNESCO for the coming years are included in the United Nations resolution and Agenda 2030 for sustainable development and that such programs aim to transform our world through improved citizenship [4]. Here, citizenship must be understood from a global point of view as a fundamental category in the conceptual framework of the internationalization of higher education. This approach is based on the fact that one of education’s purposes is to train people with the criteria and capacities to function in an increasingly multicultural and interdependent society, requiring citizens with cognitive, social, and emotional competences that will help them to value, understand, and respect current social needs; work in multicultural teams; and be able to actively and responsibly participate in the solutions to global problems [5].

Several studies have been conducted on the use of information and communication technologies (ICTs) among young people, as well as on the qualifications of young people in technology integration [6,7,8,9,10,11] at both the personal and academic level. The development of digital competence must ultimately be perceived as lifelong learning [12].

Several authors [13,14] claim that it should not be assumed that every young person has digital competence because such individuals need training in this area. Moreover, young people use limited digital tools when they start their degree studies. For Gutiérrez and Tyner, “What would be more worrying is that compulsory schooling would not fulfill its basic function of providing literacy, understood as a preparation for life in the digital society” [15].

On the other hand, ICTs have advanced in the field of communication, facilitating collaborative work and multiplying the possibilities for students and teachers to be connected via the use of virtual tools that encourage collaborative learning through blogs, websites, electronic journals, social networks, academic search engines, and platforms like MOOCs (massive online open courses). These tools will facilitate the acquisition of positive attitudes in the construction of knowledge and group cohesion, while boosting the acquisition and retention of knowledge, improving problem-solving abilities, the expression of ideas, motivation, and personal satisfaction [16], thereby generating critical thinking. With this technology, it will become easier to know and compare concepts [17,18] and thus move forward in knowledge generation, such as in new tutoring approaches [19].

Focusing on the degrees that are the object of our study, different universities have carried out studies to identify the technological competences among students in teaching degree studies, such as at the International University of Rioja, International University of Valencia, Technological University of Chile, University of Seville, University of Córdoba, University of País Vasco, the and National Central University of Taiwan, among others. The results show that automated office software, such as word processors and slideshow presentation programs are the programs known best by students [20]. Regarding of the use of blogs, wikis, or social bookmarks, most students do not know how to use the tools related to Web 2.0 [20,21]. That is, we need to go deeper into the development of ICTs and see how they generate creativity and innovation, as well as determining their possibilities when studying and carrying out academic work [22,23,24], where research, information management, and critical and creative thinking are promoted to achieve the development of digital citizenship [25,26].

Another matter of interest is the myth of “digital natives” and “digital immigrants”. Some studies have shown that digital natives—i.e., the university students—do not have strong skills in the technological tools necessary to interact in new technological environments and that digital immigrants are not technologically qualified [20,27] and should instead be considered digital students [28,29] based on the speed with which they handle some technologies from an instrumental point of view, confusing access to information with the construction of knowledge.

In addition, the higher education institute of UNESCO, in their *“COVID-19 and Higher Education: the immediate effects the day after”* report [30], in relation to the problems of students in virtual training, expressed concern about students’ communication with their peers and teachers and their connectivity with others, with the concern being lower regarding social isolation and general anxiety, but indicating low satisfaction among students with the virtual model and teaching during the COVID-19 pandemic.

The gendered differences in the use of ICTs are currently a threat of social significance [31] that must be fought by all sectors of society, including the educational field. Several studies [32,33,34] highlighted that female students are perceived as less competent in the use of ICTs than their male counterparts. This perception might have justification in the social imaginaries that are built around women in the technological field and the competences associated with the use of these tools, which have been traditionally seen as a male domain [35].

Moreover, other investigations [36,37,38] underlined some inequalities in favor of men regarding the use and knowledge of different kinds of technology, as well as a more positive attitude towards their use [39,40].

However, what are classrooms currently demanding, and how can we improve relevant strategies and engage in pedagogical innovation [4]? The birth of social networks (Instagram, Facebook, Twitter, LinkedIn, WhatsApp, YouTube, etc.) is introducing new types of profiles into educational practice and changing the relationships between teachers and students. It is possible to adapt social media services as appropriate tools in the teaching and learning process, especially to improve the interactions between teachers and students [41,42]. However, at present, in higher education, social networks are rarely used to improve student participation in collaborative learning [43,44].

Some of the perceptions that teachers have regarding social networks relate to the feeling of unsuitability of these technologies in teaching practice [45], the lack of control by teachers [46], and concern over a lack of privacy [47,48]. Other discourses on social networks revolve around their contribution to greater disconnection [49,50] or a lack of experience in their use at the institutional and academic level [47]. On the other hand, some investigations are already contributing to changing these perceptions and are focusing on the benefits of social networks and collaboration in learning and communication with students [51,52], as well as the benefits of the online work of students [53].

Social networks are a great tool to promote learning in the community, encourage the participation of students, and generate knowledge discussions [54,55,56]. Other investigations, such as those conducted by Al-Ufi and Fulton in [57] and Hamid, Waycott, Kurnia and Chang [42], have argued for the extensive benefits of social networks in higher education, as well as the possibilities for connectivity, conversation, and teamwork with such devices. All of these tools improve the satisfaction, confidence, and the participation of students [42,58]; motivate students [59]; improve learning and teacher involvement [60]; offer self-learning material [41]; and provide emotional and personal support [61]. Moreover, social networks in higher education are very valuable for improving academic performance through collaborative learning [43], where students and teachers use social networks that are interesting to them [42].

Regarding the advantages of social networks in the classroom, Buxarrais [62] proposes their use, as such tools encourage the development of attitudes and skills related to working collaboratively and more independently when searching and selecting materials. Because social networks are a part of students’ lives, the use of such tools is a regular part of each student’s day, making it easy for students to make the most of these tools; moreover, students do not feel like they are addicted to social media [63]. In addition, we found a number of concerns about the use of social networks for educational purposes, particularly as they are used as learning tools [47]. Other problems related to this technology are connected to the effects of social networks on the time dedicated to study, a loss of control [47], or the use of social networks to diffuse unrelated information [64]; therefore, institutions must propose a new pedagogical approach and define new strategies, methodologies, and tools to meet this social demand [47].

Specifically, there is an improvement in communicative processes when using YouTube to communicate [65]. Furthermore, the use of YouTube can help increase user communication and participation [66,67,68]. Moreover, teaching the use of YouTube and its practices in classrooms will contribute to the democratization of knowledge and help in the selection and reception of self-made contents. Moreover, students who are capable of creating their own audiovisual content obtain better academic results than those who are not [69].

Therefore, the acquisition of digital competence by the student body should not only be considered for adapting to the new social and labor demands of the 21st century. During the confinement caused by COVID-19, the educational community has had to deal with profound changes in a very short period of time and adapt to online teaching. This has led to high levels of stress among students, who did not know how their learning would develop in virtual environments, in addition to the emotions derived from a lack of social contact and confinement. In this way, the acquisition of good technological skills could help alleviate the negative effects derived from concern about how the teaching–learning process will be developed in online contexts. Thus, the objective of this study is to analyze the influence of the use of digital tools and social networks of university students during the COVID-19 pandemic.

## 2. Methods

To respond to the needs of our study, we chose to design non-experimental research, since the main objective of the investigation was to “analyze the impact of digital tools and social media on the development of digital competence of university students during COVID19 pandemic” [70]. To this end, we focused on the three key elements of any research process: the data collection instrument, the participants and application context, and the procedure to be used.

### 2.1. Population and Sample

The sample selected included a total of 581 university students enrolled in Childhood Education, Primary Education, and Social Education degrees during the 2019/2020 academic year at the University of Jaen.

In total, 81.4% (472) of the subjects were women, and 18.6% (108) were men. The mean age was about 21 years old, with 82.5% of the sample located in the age interval between 18 and 23 years old. Regarding the degree the students were enrolled in, 65.1% of the participants were studying a Childhood Education degree, 18.1% a Primary Education Teaching degree, and the remaining 16.8% a Social Education degree. Regarding the subjects in which they were enrolled, 58.5% were enrolled in School Center Organization in Infant Education; 14.2% in Primary Education Organization; 12.3% in the Design, Development, and Evaluation of Social Education Programs; 10.2% in Educational Multimedia in Infant and Primary Education; and 4.7% in Diagnosis and Evaluation in Social Education.

Lastly, the vast majority of subjects (99.5%) had a computer or a tablet, and 98.4% had an Internet connection at home and mainly connected from home (69%). Likewise, the majority, 87.7%, were trained in the use of ICTs primarily offered by the University (82.5%). In total, 51.4% of the surveyed students spent between 4 and 9 h a week dedicated to the use of ICTs related to their studies.

The type of sampling used was non-probabilistic, casual, and accidental sampling, where the investigator directly and intentionally selects the sample, mainly because the sample is easily accessible and representative of the population [71].

### 2.2. Data Collection Procedure and Instrument

In this work, we used a descriptive quantitative methodology through a survey. We designed an ad hoc questionnaire as an information collection instrument, which is one of the most commonly used techniques in investigations in the field of digital competence [72]. This survey used a Likert-type scale where subjects had to indicate, on a 1–10 scale, their digital competence degree, where a value of 1 means that the individual feels completely unable to perform the task, and 10 means that the individual has mastered it completely. The instrument was adapted from Gutiérrez, Cabero, and Estrada [1]. The structure of the questionnaire is as follows:

- Dimension 1. Technological literacy: 13 items.
- Dimension 2. Search and information processing: 6 items.
- Dimension 3. Critical thinking, problem solving and decision making: 4 items.
- Dimension 4. Communication and collaboration: 9 items.
- Dimension 5. Digital citizenship: 6 items.
- Dimension 6. Innovation and creativity: 6 items.

For the participants to complete the questionnaire, the students were sent a link to the instrument to fill in online, as well as the procedural clarifications, ensuring at all times the confidentiality and anonymity of the data collected. The instrument ultimately generated a record of the responses prepared for their statistical interpretation and analysis. This instrument was developed through the Google forms tool, which allows us to send it en masse and receive the data online.

### 2.3. Data Analysis

Analysis of the available information was carried out through two different stages. First, we carried out an exploratory factor analysis (EFA) to determine the subjacent factorial structure of the Digital Competence in Higher Education Students scale (DCHES). The required analyses were carried out using the STATA statistical package, version 15 [73,74,75]. Following Costello and Osborne [76], as the method for extracting the factors, we selected a true method of factor analysis, discarding the principal component analysis approach that is commonly used by default in various statistical packages. To choose among the alternative methods available (unweighted least squares, generalized least squares, maximum likelihood, principal axis factoring, alpha factoring, and image factoring), we followed the recommendations of Frabigar [77] and examined whether it is possible to assume the hypothesis of multivariate normality required to use the maximum likelihood extraction method. This is important because this procedure can produce misleading results when assumptions of multivariate normality are severely violated.

In the second phase, we used SEM (structural equation modeling), a statistical approach, to test the relationships among observed and latent variables [78]. More concretely, we estimated a multiple indicators multiple causes (MIMIC) model (latent variable model with multiple indicators) using the Mplus 8.4 program [79]. Within the framework of structural equation modeling (SEM), a MIMIC (multiple indicators multiple causes) model features one or more latent variables that are predicted by observed variables or covariates [80]. Thus, a MIMIC factor includes both cause indicators and effect indicators [81].

### 2.4. Exploratory Factor Analysis (EFA) Results

We began our analysis determining if the data was adequate to be analyzed using an EFA approach. For this purpose, two measures for the adequacy of the sample are usually used: the Kaiser–Meyer–Olkin measure of sampling adequacy (KMO), which indicates the proportion of variance in variables that might be caused by underlying factors, and Bartlett’s test of sphericity, which tests the hypothesis that a correlation matrix is an identity matrix [82]. We used the *factortest* command available in STATA to calculate these measures [83]. The results of both demonstrated that our data met the factor analysis criteria: the KMO measure was 0.959, and Bartlett’s test was statically significant, with χ^2^_(946)_ = 11,714.08 and *p* < 0.001.

To determine whether the data followed a normal multivariate distribution, we used the *mvtest* command available in STATA to calculate the Doornik–Hansen test of multivariate normality [84]. The obtained results indicated that the data could not be assumed to be distributed according to a multivariate normal, so the ML method was discarded to extract the factors (χ^2^_(88)_ = 1085.34; *p* = 0.000). Thus, we choose to use principal axis factoring as the method of extraction [76,77]. Moreover, because the factors could be correlated from a theoretical point of view, as a rotation method, we chose Oblimin with Kaiser normalization.

**Figure 1:**
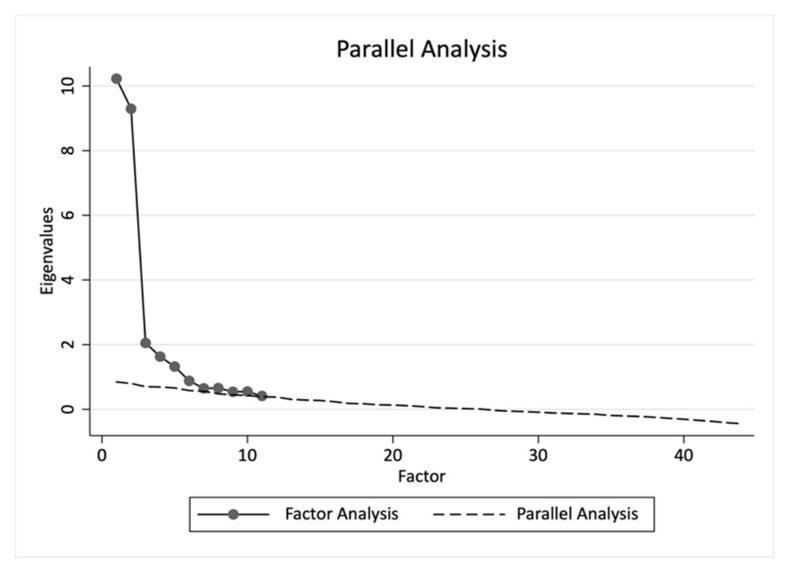
Result of Horn’s parallel analysis using the *fapara* command.

On the other hand, one of the most important decisions in factor analysis is how many factors to retain. Although a commonly utilized criterion is retaining factors whose eigenvalues are greater than unity [84,85], there are various problems associated with this approach, and thus its application is not recommended [86,87]. In accordance with the recommendations of Velicer [88], we used Horn’s parallel analysis to determine the number of factors to retain. The required analyses were carried out in the statistical package STATA using the *fapara* command [89]. The results obtained after the analysis indicated the desirability of retaining seven factors. In Figure 1, the dashed line intersects the solid line for the first time at the point that represents the extracted seventh factor.

Thus, our solution retained seven factors, which explained 58.9% of the variance. Table 1 shows information for interpreting the factors extracted. For oblique rotations, where the factors are allowed to correlate, we obtained a solution with various matrices. The pattern matrix that holds the loadings essentially presents a regression equation where the standardized observed variable is expressed as a function of the factors (loadings are regression coefficients).

To evaluate the reliability of each subscale, we used two indicators. First, the Cronbach’s alpha coefficient (α) is often used as a measure of the internal consistency of a test or scale, varying the acceptable values between a minimum of 0.7 and a maximum of 0.95 [90]. Given that the use of this coefficient is not exempt from criticism [61,91], we also calculated the omega (ω) coefficient [91], which has been proposed as an alternative to overcome some of the disadvantages inherent to the Cronbach’s alpha coefficient [92]. To calculate these coefficients (ω; α) and their corresponding confidence intervals at 95%, we used the R Statistical Package version 4.0.2 (R Core Team, Vienna, Austria) and “userfriendlyscience” library [93].

Thus, our instrument was structured into two large blocks. This structure was intended to characterize the participants in this research (by gender, age, degree, subject, contextual data, participatory methodologies, and attitudes towards ICTs); secondly, it consisted of 44 items on the study of the Competition Higher Education Students’ Students and was set up by a first factor (DIM1) and integrated by 11 items related to the searching, processing, resolution, and communication of information. The second factor (DIM2) consists of five items that relate to the technological literacy of the respondents. The third factor (DIM3) reflects the effects of those items on the use of ICTs and consists of four elements. The fourth factor (DIM4) includes items related to collaboration between the people participating in the study and is composed of four other items. The fifth factor is composed of three items and represents the degree of digital citizenship of the respondents (DIM5). The sixth factor (DIM6) is composed of seven indicators and is related to the digital performance of the respondents, while the seventh (DIM7) is labeled as “leadership, innovation and creativity” and is composed of nine items.

As shown in Table 1, the different results of EFA show adequate levels of internal consistency, with the values for the Cronbach’s alpha and McDonald omega above the minimum recommended values in the literature. Following Bandalos and Gerstner [94] and Hair et al. [86], we consider that the pattern coefficients have practical significance as long as their minimum value is in the range of 0.30–0.40 in absolute terms. Table 1 shows the correlations between the different subscales obtained and Table 2 presents the component correlation matrix.

### 2.5. The MIMIC Model: Results

**Figure 2:**
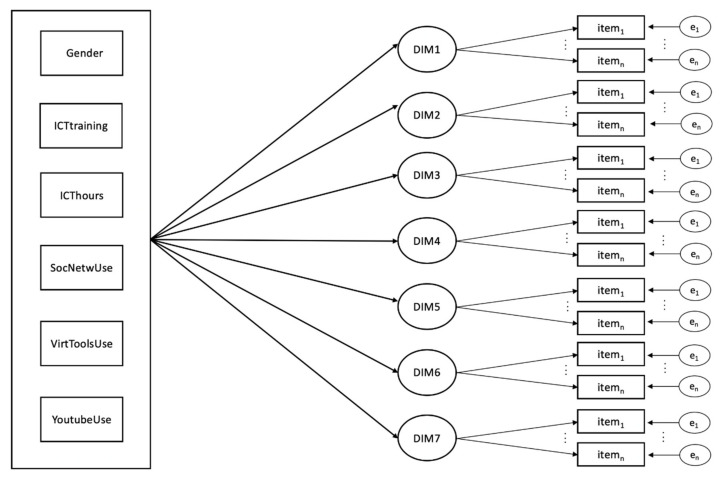
Multiple indicators multiple causes (MIMIC) model to be tested.

The MIMIC model represented in Figure 2 illustrates the hypothesized relationship between the various variables of interest and the dimensions of DCHES. In this model, every dimension of DCHES is measured through the indicators that were determined by the previous EFAs (see Table 1). We also consider six covariates that, according to the literature, can influence these dimensions. These covariates include gender (with 0 = woman and 1 = man), ICT training (previous training in the field of ICT, where 0 = no and 1 = yes), ICThours (number of hours that information and communication technologies were used to study), SocNetwUse (degree of the use of social networks to carry out the work commissioned by teachers, with a Likert-type scale from 1 to 10), VirToolsUse (degree of the use of virtual tools to develop teamwork, with a Likert-type scale from 1 to 10), and YoutubeUse (YouTube’s degree of use to communicate; Likert-type scale from 1 to 10).

Since the model considers both ordered categorical and categorical measures, we use the WLSMV estimation method (robust weighted least squares), which is a robust estimator recommended in such a situation [95,96]. The WLSMV estimator was developed by Muthén, du Toit, and Spisic [97], and was designed specifically for use with small and moderate sample sizes.

## 3. Results

After estimating the model, and before analyzing the possible conclusions derived from the results obtained, we verified that the goodness of fit was adequate. Following the recommendations of West [95], we confirmed that the model provided an adequate fit to the data (χ^2^ = 2693.744, df = 1051, *p* < 0.01, χ^2^/df = 2.5; RMSEA (Mean Square Approximation Error) = 0.06, SRMR (Standarized Root Mean-Square) = 0.04, CFI (Comparative Fit Index) = 0.959, TLI (Tucker-Lewis Index) = 0.953). Next, we checked the validity and reliability of the different measurement scales linked to the dimensions considered. Table 3 shows the standardized coefficients of the measurement models and values of composite reliability (CR), as well as the average variance extracted (AVE) for each scale measurement. Composite reliability is an indicator of internal consistence, and values of 0.7 or more are recommended by the literature. On the other hand, the average extracted variance allowed us to evaluate the convergent validity, using 0.5 as the minimum desirable value for this indicator, as per the literature. As can be seen in Table 4, the values calculated for these two indicators exceeded the recommended minimums, so the scale is valid and reliable [81,84].

Finally, Table 5 presents the estimated coefficients that show the relationship between the covariates considered in the model and the seven dimensions of the DCHES scale during COVID-19.

The results obtained show the importance of two of the covariates considered in our study in explaining the variability of the different dimensions of the DCHES scale analyzed. In this sense, both the degree to which virtual tools are used to develop teamwork (VirToolsUse) and the degree of use of YouTube when communicating (YoutubeUse) most fully explained the level of digital skills among the university students studied.

## 4. Discussion

The world has experienced profound transformations since the global pandemic caused by COVID-19. In recent months, all the systems that comprise society have been affected in all countries. While educational changes certainly occur more slowly than changes in other individual contexts, the pandemic has shown the potential for change and adaptation among human beings. This capability became clear after the strict lockdown decree to which we were subjected in Spain in the middle of March. In barely forty-eight hours, face-to-face educational institutions became virtual, along with the resulting changes for the educational community.

While university institutions may have had fewer infrastructure and resource problems when making this change thanks to virtual campuses, agreements with technology companies for the provision of services, etc., we must also consider other factors emerging from the lockdown, such as the radical transformation experienced without sufficient time to adjust and develop an efficient teaching–learning process, the need for the competent use of ICTs, as well as the fear and concern over health and decrease in social relations, among others.

In this way, digital competence is a fundamental key element of present-day study. This dimension relates to the ability of the individual to engage in responsible use of the Internet, focusing on communication, socialization, and learning [1,3].

Our research focused on analyzing the influence of the use of digital tools and social media on the digital competence of higher-education students during the COVID-19 pandemic. Our model incorporated variables that, according to the literature reviewed, could have an impact on the digital competence of students. Of the variables analyzed, our results indicate that gender is only relevant for two of the seven dimensions considered (the searching, processing, resolution and communication of information (DIM1) and digital performance (DIM6)). In both cases, men showed a higher level of competence in these dimensions compared to women. These results coincide with those of other studies that describe women as ICT users in increasingly similar numbers to those of men [98]. However, these studies agree that there are still inequalities in the use and knowledge of different types of technology in ICT training and in the competences needed to live and work in environments underpinned by those technologies [33]. Gender equality in ICT management is, therefore, considered necessary for the initial training of higher education students [99].

The results obtained indicate that previous training in ICTs only affects the competences shown by students related to the use of devices and the Internet (DIM3). The number of hours that ICTs are used by students in their education is relevant when determining the level of technological literacy of the students (DIM2), as well as the creative thinking, innovative processes, and leadership capacity of students due to the use of ICT (DIM7).

On the other hand, the degree to which social networks are used to develop the work commissioned by students did not seem to be an important factor when analyzing the digital competences of the students participating in the survey. This variable only positively affected the use of the means and resources for communication and security in the use of technologies (DIM4), which agrees with other studies [100], but the remaining dimensions were not significantly affected.

Finally, among the variables considered in our model as potential determinants for the social–emotional wellbeing of students during lockdown, two stand out for having a positive and statistically significant influence on all dimensions. Both the degree to which virtual tools are used to develop teamwork [42,51,52,53,57,68] and the degree of the use of YouTube to communicate [65,66,67] were shown to have a positive impact on all dimensions considered. Our results indicate that to improve the digital literacy of university students, it would be of interest to promote the use of YouTube as a teaching tool, as well as the use of other virtual tools, for developing teamwork among students [54,55,56].

Digital tools and social media during lockdown in Spain have demonstrated that learning is not exclusively developed in formal spaces established for that purpose, whether face-to-face or virtual. The crisis caused by COVID-19 has facilitated the creation of alternative and varied environments to search for information, consume content, and create and share content, as well as other factors that enable greater communication, socialization and networking for collaborative work.

## 5. Conclusions

This study serves as a starting point to open new lines of research, such as those related to the socio-emotional well-being of students under online teaching, since a significantly higher level of fear, anger and impotence related to technology is observed [100]. 

The limited social sharing that resulted from the COVID-19 pandemic could also foster negative emotions. There is evidence that social isolation can, moreover, trigger stress-related emotions and reduce well-being [101,102]. Likewise, the authors in [103] observed effects on students’ support from the school administration, their self-efficacy with computers, and their relationships with their partners in the face of the stress caused by technology. Indeed, “Keeping the pulse on students’ emotional health” is one of the four challenges identified by the OECD (Organisation for Economic Co-operation and Development) in promoting digital learning and online collaboration [104]. Technology can be a tool, but it cannot replace face-to-face interaction [105]. Aside from the social component, there are many other factors that explain how and why students experience and appreciate online courses. For example, students’ experiences with e-learning are related to their overall satisfaction with life [104]. Digital readiness and a material-rich online learning environment also contribute to student well-being [106].

Finally, certain limitations in the study should be considered. For example, there is a need to further explore the acquired quantitative data with a qualitative analysis to complement these results with students’ perceptions of this change in modality from face-to-face to online learning. For future research, it would be useful to carry out longitudinal studies to observe the evolution of the study topic, from where it started to the current state of the issue, as it has been a year since the pandemic was declared, and there have been several restrictive measures and changes in the educational environment.

## Figures and Tables

**Table 1:** Results of exploratory factor analysis (EFA): factor loading using principal axis factoring with direct Oblimin rotation (pattern matrix coefficients).

Factors and Items	Reliability Indicators	F1	F2	F3	F4	F5	F6	F7
**DIM1. Search, processing, resolution, and communication of information**	ω = 0.932 CI: [0.922, 0.943] α = 0.932 CI: [0.922, 0.942]							
I am able to locate information through different sources and databases available on the Internet (DCHES14).		0.414						
I can identify relevant information by evaluating different sources and their provenance (DCHES15).		0.618						
I am able to organize, analyze and ethically use information from a variety of sources and media (DCHES16).		0.582						
I synthesize selected information appropriately for the construction and assimilation of new content, using tables, graphs or diagrams. (DCHES17).		0.541						
I plan information searches for problem solving (DCHES19).		0.601						
I am able to identify and define problems and/or research questions using ICT (DCHES20).		0.530						
I use digital resources and tools to explore current world issues and solve real problems, addressing personal, social, professional needs… (DCHES21).		0.519						
I can analyze the capabilities and limitations of ICT resources (DCHES22).		0.524						
I share information of interest with my peers using a variety of digital environments and media (DCHES24).		0.443						
I effectively communicate information and ideas to multiple audiences, using a variety of media and formats (DCHES25).		0.483						
I am able to develop cultural understanding and global awareness through communication with other students and professionals from other cultures (DCHES26).		0.501						
**DIM2. Technological literacy**	ω = 0.858 CI: [0.836, 0.881] α = 0.855 CI: [0.832, 0.878]							
I am proficient in different office tools for information processing, such as word processors, spreadsheets, databases… (DCHES4).			0.464					
I investigate and solve problems in systems and applications (configure e-mail, configure antivirus, defragment the hard disk, etc.) (DCHES5).			0.525					
I am able to use different image, audio or digital video processing tools (DCHES6).			0.664					
I can design web pages using software, including text, images, audio, links, etc. (DCHES9).			0.465					
I know how to use collaborative work software using online tools such as Groupware (Google Apps, BSCW, OpenGroupWare…) (DCHES11).			0.548					
**DIM3. ICT use**	ω = 0.758 CI: [0.719, 0.789] α = 0.748 CI: [0.708, 0.787]							
I am able to use different types of operating systems installed on a computer (Microsoft Windows, Linux, Mac…) and on mobile devices (iOS, Android, BlackBerry OS…) (DCHES1)				0.631				
I am able to use different mobile devices (Smartphone, Tablet, PDAs…) (DCHES2).				0.767				
I surf the Internet with different browsers (Internet Explorer, Mozilla Firefox, Safari, Opera…) (DCHES3).				0.416				
I feel competent to use the virtual management (virtual secretary, library services, etc.) of my university (DCHES13).				0.509				
**DIM4. Communication and collaboration**	ω = 0.768 CI: [0.729, 0.806] α = 0.772 CI: [0.736, 0.809]							
I can communicate with other people using synchronous communication tools via the web (chat, instant messaging services, Skype…) (DCHES7).					0.405			
I am able to communicate with others using asynchronous web-based communication tools (forums, social networks, mailing lists) (DCHEES8).					0.425			
I am able to coordinate group activities using online tools and media (DCHES28).					0.359			
I interact with other colleagues and users using social networks (Facebook, Ning, Twitter…) and communication channels (Blog, YouTube channel…) based on ICT (DCHES29).					0.412			
**DIM5. Digital citizenship**	ω = 0.857 CI: [0.832, 0.882] α = 0.856 CI: [0.831, 0.881]							
I am ethically committed to the use of digital information and ICT, including respect for copyright, intellectual property and proper referencing of sources (DCHES33).						0.585		
I promote and practice safe, legal and responsible use of information and ICT (DCHES34).						0.632		
I demonstrate personal responsibility for lifelong learning using ICTs (DCHES35).						0.654		
**DIM6. Digital performance**	ω = 0.873 CI: [0.854, 0.893] α = 0.869 CI: [0.849, 0.889]							
I know how to use collaborative work software using online tools such as Groupware (Google Apps, BSCW, OpenGroupWare…) (DCHES10).							0.412	
I use graphic organizers and software for making concept and mind maps (CmapTool, Mindomo, etc.), diagrams or schemes, to present the relationships between ideas and concepts. (DCHES18).							0.629	
I configure and troubleshoot hardware, software and networking systems to optimize their use for learning and productivity (DCHES23).							0.408	
I can use software (SlideShare, Google Docs, etc.) and technological tools to manage and communicate information with colleagues and other online users (DCHES27)							0.352	
I am able to manage professional networks (Linkedin, etc.) (DCHES30).							0.696	
I am able to design, create or modify a Wiki (Wikispaces, Nirewiki, etc.) (DCHES31).							0.669	
I can use social bookmarking to locate, store and tag Internet resources (DCHES32).							0.511	
**DIM7. Leadership, innovation and creativity**	ω = 0.924 CI: [0.913, 0.936] α = 0.924 CI: [0.913, 0.935]							
I consider myself competent to make constructive criticisms, judging and making contributions to the ICT work developed by my colleagues (DCHES36).								0.422
I exercise leadership for digital citizenship within my group (DCHES37).								0.506
I exhibit a positive attitude towards the use of ICTs to support collaboration, learning and productivity (DCHES38).								0.532
I have the ability to come up with original, novel and useful ideas using ICT (DCHES39).								0.688
I am able to create original work using traditional and emerging ICT resources (DCHES40).								0.654
I identify trends by anticipating the potential uses that ICT can lend me (DCHES41).								0.680
I use models and simulations to explore complex systems and issues using ICTs (DCHES42).								0.548
I develop materials where I use ICT in a creative way, supporting the construction of my knowledge (DCHES43).								0.529
I am able to adapt to new situations and technological environments (DCHES44).								0.388

**Table 2:** Component correlation matrix.

Factor	1	2	3	4	5	6	7
**DIM1**	1000						
**DIM2**	0.444	1000					
**DIM3**	0.317	0.463	1000				
**DIM4**	0.329	0.352	0.340	1000			
**DIM5**	0.412	0.271	0.308	0.305	1000		
**DIM6**	0.467	0.449	0.233	0.274	0.188	1000	
**DIM7**	0.478	0.348	0.217	0.436	0.385	0.389	1000

Extraction Method: Principal Component Analysis. Rotation Method: Oblimin with Kaiser Normalization.

**Table 3:** Standardized solution using the robust weighted least squares (WLSMV) estimation method.

Dimension	Item	Standardized Solution	AVE and CR
DIM1	**DCHES** 14	0.770	CR = 0.9402 AVE = 0.6117
	**DCHES** 15	0.712	
	**DCHES** 16	0.793	
	**DCHES** 17	0.772	
	**DCHES** 19	0.820	
	**DCHES** 20	0.780	
	**DCHES** 21	0.821	
	**DCHES** 22	0.740	
	**DCHES** 24	0.806	
	**DCHES** 25	0.800	
DIM2	**DCHES** 4	0.741	CR = 0.8826 AVE = 0.6016
	**DCHES** 5	0.759	
	**DCHES** S6	0.822	
	**DCHES** 9	0.707	
	**DCHES** 11	0.841	
DIM3	**DCHES** 1	0.814	CR = 0.8342 AVE = 0.5584
	**DCHES** 2	0.743	
	**DCHES** 3	0.661	
	**DCHES** 13	0.763	
DIM4	**DCHES** 7	0.690	CR = 0.8049 AVE = 0.5101
	**DCHES** 8	0.639	
	**DCHES** 28	0.827	
	**DCHES** 29	0.687	
DIM5	**DCHES** 33	0.862	CR = 0.8929 AVE = 0.7358
	**DCHES** 34	0.810	
	**DCHES** 35	0.899	
DIM6	**DCHES** 10	0.663	CR = 0.8924 AVE = 0.5441
	**DCHES** 18	0.681	
	**DCHES** 23	0.787	
	**DCHES** 27	0.811	
	**DCHES** 30	0.664	
	**DCHES** 31	0.742	
	**DCHES** 32	0.798	

**Table 4:** Standardized solution using the WLSMV estimation method II.

Dimension	Item	Standardized Solution	AVE and CR
DIM7	**DCHES** 36	0.779	CR = 0.9407 AVE = 0.6394
	**DCHES** 37	0.675	
	**DCHES** 38	0.773	
	**DCHES** 39	0.820	
	**DCHES** 40	0.886	
	**DCHES** 41	0.862	
	**DCHES** 42	0.826	
	**DCHES** 43	0.830	
	**DCHES** 44	0.723	

**Table 5:** Coefficients estimated for the covariates (standardized solution using the WLSMV estimation method).

	DIM1	DIM2	DIM3	DIM4	DIM5	DIM6	DIM7
**Gender**	0.119 **	n.s.	n.s.	n.s.	n.s.	0.107 *	n.s.
**ICTtraining**	n.s.	n.s.	0.111 *	n.s.	n.s.	n.s.	n.s.
**ICThours**	0.091 ^+^	0.137 **	n.s.	n.s.	n.s.	n.s.	0.132 *
**SocNetwUse**	n.s.	n.s.	n.s.	0.105 *	n.s.	n.s.	n.s.
**VirtToolsUse**	0.290 **	0.177 **	0.280 **	0.342 **	0.315 **	0.139 **	0.269 **
**YoutubeUse**	0.217 **	0.248 **	0.158 **	0.177 **	0.123 *	0.240 **	0.203 **

** *p* < 0.01 * *p* < 0.05 ^+^ *p* < 0.1 (two-tailed); n.s. = not significant

## Data Availability

The data presented in this study are available on request from the corresponding author. The data are not publicly available due to this study belongs to an ongoing research project.
